# Saturated fatty acid-crystals activate NLRP3 inflammasome

**DOI:** 10.18632/aging.101892

**Published:** 2019-03-26

**Authors:** Tadayoshi Karasawa, Masafumi Takahashi

**Affiliations:** 1 Division of Inflammation Research, Center for Molecular Medicine, Jichi Medical University, Tochigi, Japan

**Keywords:** cytokine, inflammation, innate immunity, interleukin-1, macrophage

The deposition of crystals including cholesterol and calcium phosphate is a hallmark feature of atherosclerotic lesion. The deposited crystals have recently been shown to be recognized as danger signals by pattern recognition receptors (PRRs) and be involved in the initiation of inflammatory responses during the development of atherosclerosis [1]. As PRRs, nucleotide–binding oligomerization domain-like receptor (NLR) family, pyrin domain containing 3 (NLRP3) has emerged as a key player for the crystal-induced inflammation in atherogenesis. NLRP3 is mainly expressed in innate immune cells, such as macrophages, and forms a molecular complex called NLRP3 inflammasome which is composed of NLRP3, apoptosis-associated speck-like protein containing a caspase recruitment domain (ASC), and cysteine protease caspase-1 [2]. When cells are exposed to danger signals, the components of NLRP3 inflammasome assembles and activates caspase-1, leading to the subsequent processing of pro-IL-1β into its mature form to initiate inflammatory responses [2].

Saturated fatty acids (SFAs) are associated with increased risk of atherosclerosis, and has been regarded as a danger signal which activates several PRRs to induce inflammatory response in metabolic diseases [3]. For instance, SFAs have been shown to activate Toll-like receptor 4 (TLR4), a well-known PRR, and induce the expression of inflammatory cytokines [3]. Recent investigations also suggested that SFAs activate NLRP3 inflammasome by generation of mitochondrial reactive oxygen species (ROS) via dysregulated mitophagy [4]; however, the precise mechanism by which SFAs trigger NLRP3 inflammasome activation remained unclear. We have recently identified a novel mechanism underlying SFAs-induced NLRP3 inflammasome activation in macrophages [5].

**Figure 1:**
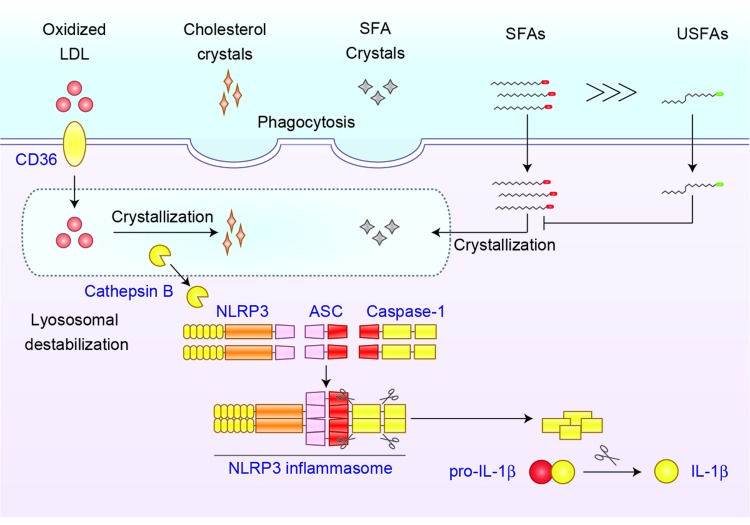
**NLRP3 inflammasome activation mediated by lipid crystals.** Soluble lipids form crystals to activate NLRP3 inflammasome. The imbalance between SFAs and USFAs causes intracellular crystallization of SFAs. Similarly, oxidized LDL promotes formation of cholesterol crystals. Intracellularly formed or phagocytosed cholesterol crystals and SFA crystals cause lysosomal destabilization, which induces the leakage of lysosomal enzyme cathepsin B, resulting in the activation of NLRP3 inflammasome.

Although the upstream mechanisms of NLRP3 inflammasome have not been fully understood, several common upstream pathways for NLRP3 inflammasome activation are identified: potassium (K^+^) efflux, generation of mitochondrial ROS, and lysosomal rupture-induced cathepsin release. Of these, it has been well accepted that lysosomal rupture-induced cathepsin release is responsible for NLRP3 inflammasome activation by particulate matters, such as monosodium urate, cholesterol crystals, and calcium pyrophosphate dihydrate [1]. We found that NLRP3 inflammasome activation by SFAs, such as palmitic acid and stearic acid, are inhibited by the inhibition of lysosomal cathepsin B, indicating the involvement of particulate matters. Further, intracellular crystals are clearly detected in the SFA-loaded macrophages. The crystallization occurs inside the cells because the phagocytosis inhibitor cytochalasin D failed to inhibit SFA-induced NLRP3 inflammasome activation. Similarly, oxidized low-density lipoprotein has shown to be incorporated by macrophages via scavenger receptor CD36 and cause crystallization, resulting in NLRP3 inflammasome activation [6]. Because CD36 also acts as FA transporter, we assume that SFAs are incorporated by macrophages via CD36, at least in part. Another important issue to be addressed is that SFA-induced crystallization and NLRP3 inflammasome activation are inhibited in the presence of unsaturated fatty acids (USFAs). These results suggest that excess SFAs induce an imbalance of cellular fatty acid composition, resulting in fatty acid-derived crystallization and NLRP3 inflammasome activation (Figure 1).

We further demonstrated that excess SFAs induce crystallization and IL-1β release not only in macrophages *in vitro* and but also in mice *in vivo*. Administration of ethyl palmitate emulsion, which is metabolized to palmitic acid after hydrolysis, induces crystal deposition and neutrophil accumulation into the spleen. We also confirmed that SFA-derived crystals promote neutrophil recruitment in an IL-1β-dependent manner. These findings indicate the role of SFA-derived crystals in acute inflammation; however, the role of neutrophil accumulation in the progression of atherosclerosis remains unclear. In this regard, Warnatch et al. [7] recently reported that cholesterol crystals induce formation of neutrophil extracellular traps (NETs) to amplify inflammatory responses in the atherosclerotic lesion. Moreover, the isolated NETs prime IL-1β transcription in macrophages to enhance inflammasome activation. Based on these findings, we assume that SFA-derived crystals can induce NETs formation which enhances NLRP3 inflammasome activation in infiltrated macrophages. Further investigations are necessary to understand the precise role of fatty acid-derived crystals and NLRP3 inflammasome in the pathogenesis of metabolic diseases and to clarify NLRP3 inflammasome as a potential therapeutic target.
